# Microtechnology-based methods for organoid models

**DOI:** 10.1038/s41378-020-00185-3

**Published:** 2020-10-05

**Authors:** Vanessa Velasco, S. Ali Shariati, Rahim Esfandyarpour

**Affiliations:** 1 Biochemistry Department, Stanford University, Palo Alto, CA USA; 2 Department of Biomolecular Engineering, Institute for the Biology of Stem Cells, University of California, Santa Cruz, CA USA; 3 Department of Electrical Engineering, University of California, Irvine, CA USA; 4 Department of Biomedical Engineering, University of California Irvine, Irvine, CA USA; 5 Henry Samueli School of Engineering, University of California, Irvine, CA USA

**Keywords:** Bionanoelectronics, Electrical and electronic engineering

## Abstract

Innovations in biomaterials and stem cell technology have allowed for the emergence of novel three-dimensional (3D) tissue-like structures known as organoids and spheroids. As a result, compared to conventional 2D cell culture and animal models, these complex 3D structures have improved the accuracy and facilitated in vitro investigations of human diseases, human development, and personalized medical treatment. Due to the rapid progress of this field, numerous spheroid and organoid production methodologies have been published. However, many of the current spheroid and organoid production techniques are limited by complexity, throughput, and reproducibility. Microfabricated and microscale platforms (e.g., microfluidics and microprinting) have shown promise to address some of the current limitations in both organoid and spheroid generation. Microfabricated and microfluidic devices have been shown to improve nutrient delivery and exchange and have allowed for the arrayed production of size-controlled culture areas that yield more uniform organoids and spheroids for a higher throughput at a lower cost. In this review, we discuss the most recent production methods, challenges currently faced in organoid and spheroid production, and microfabricated and microfluidic applications for improving spheroid and organoid generation. Specifically, we focus on how microfabrication methods and devices such as lithography, microcontact printing, and microfluidic delivery systems can advance organoid and spheroid applications in medicine.

## Organ-on-a-Chip: Micromanufacturing offers a boost to three-dimensional cell cultures

Microtechnology-based approaches could overcome the limitations of current three-dimensional cell and tissue culture processes. Complex 3D cultures provide deeper insights into human biology and pathology than standard 2D cultures, but their high complexity brings issues like low reproducibility and low throughput. In this paper, Rahim Esfandyarpour, PhD, Assistant Professor of Electrical Engineering, & Biomedical Engineering, and his collaborators from the University of California, Irvine, Santa Cruz, and Stanford University introduce the benefits of microscale technologies. The team describe how microcontact printing of cell-scaffold proteins onto culture mediums allows for higher throughput. The team also describe the construction of an “organ-on-a-chip,” where cultures are constrained between microfluidic nutrient exchange channels, driving the development of complex and accurate tissue structures. Organ-on-a-chip devices are highly economical and offer a platform that is yet to be fully exploited.

## Introduction

**Fig. 1:**
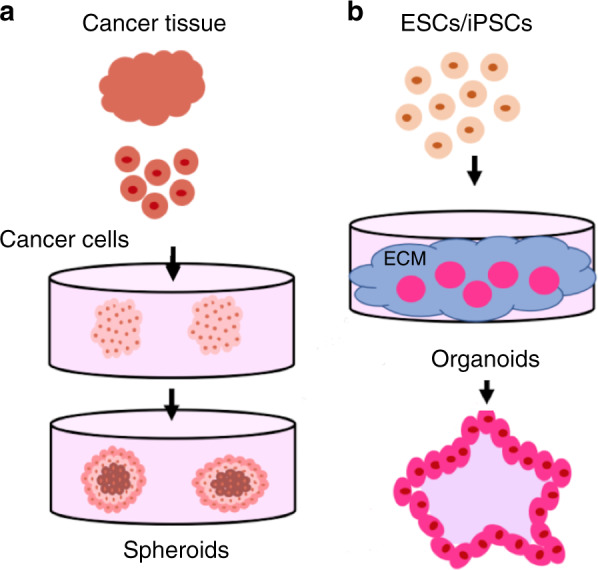
Diagram of spheroid and organoid development. **a** From human cancer tissue, tumor cells can be isolated and placed in culture to yield spheroids. **b** Embryonic stem cells (ESCs) and induced pluripotent stem cells (iPSCs) are two common stem cell types used as the cellular source for organoid production. Both ESCs and iPSCs can form a variety of organoid models when given the right signaling cues and ECM

Animal models and conventional two-dimensional (2D) cell culture models have long been used to understand human physiology and pathology^1^. Though these models have propagated numerous scientific advances, their application in modeling human physiology and pathology is limited. Animal models are inherently limited in mimicking human-specific biology due to the existing physiological differences between humans and animals. While a monolayer culture of human cells can be a window to human-specific biology, the simplicity of 2D cell culture does not reflect the complexity and cellular diversity of the tissues in vivo. In addition, our access to adult or human embryonic tissues is minimal due to ethical considerations. These limitations have led to advancements in materials and manufacturing techniques combined with stem cell technology to generate 3D human tissue-like models known as organoids and spheroids^2,3^. Organoids are three-dimensional cell culture models that self-organize into complex organ-like tissues^4^. Spheroids are 3D culture systems that can be used to model multicellular tumors; more broadly, spheroids can be defined as cell aggregates cultured on nonadherent substrates^5,6^. Consequently, they have become groundbreaking systems to study human development, disease progression, and treatment, as well as to develop personalized medicine approaches that are not possible with animal models. Typically, spheroids are formed from cancer cell lines or dissociated cell clusters from tumor tissue in nonadherent substrates (Fig. 1a)^6^. Even though organoid models can be generated from mince tissue containing epithelial cells, a large number of organoid model protocols use stem cells as the cellular source for organoid production (Fig. 1b). Stem cells are a particular type of cell and are defined by their ability to self-renew as well as their potential to make more specialized cell types. Strikingly, stem cells can give rise to differentiated progenies that self-organize into tissues that recapitulate the form and functions of the organ^7^. The cells do so by autocrine and paracrine signaling as well as via exposure to a specific extracellular matrix (ECM)^8^. Though several spheroids and organoid production techniques have been introduced recently, there are still some challenges to overcome in their production (Table 1). In particular, the reproducible production of organoids remains challenging, as their production is a complex multistep procedure that depends on multiple variables such as cell type, cellular state, and growth^7^. Spheroid production is hindered by the lack of size uniformity^9^. For instance, spheroids can range from 65 to 300 µm in size when generated by spinner flasks^10–13^. Microfabricated and microscale platforms (e.g., microfluidics and microprinting) have shown promise to address some of the current limitations in both organoid and spheroid generation^14–16^. In this review, we will discuss stem cell types, traditional techniques used for the generation of human organoid and spheroid models and their shortcomings. Our primary focus is on emphasizing state-of-the-art microtechnology-based platforms for the production of organoids and spheroids, their advantages and applications in microfabricated and microfluidic-assisted spheroid and organoid models.

**Table 1:** Comparison table of different organoid and spheroid production methods

	Extracellular Matrix Scaffold	Spinning Bioreactor	Hanging-Drop	Low-adherent Substrates	Magnetic Levitation	Bioprinted	Micropatterning	Microfluidic
**3D Culture Yield**	Organoid	Organoid and Spheroid	Spheroid	Spheroid	Spheroid	Organoid	Spheroid	Organoid
**Description**	Stem cells are placed in Matrigel (or ECM mix) and maintained in culture	Suspension cultures placed within spinner flasks or bioreactors with high-viscosity reagents	Cells are suspended in media droplet, within droplet cells aggregate at the air–liquid interface	Cell seeded onto low-adherent/hydrophilic substrates to form cell aggregates	Nanoparticles ingested by cells, cells are placed in a low-adherent substrate, and a magnet lid is used to aggregate the cells	Additive manufacture of ECM, cytokines, & cells	Microcontact printing and soft-lithography patterning of ECM	Micron-sized structures to hold 3D culture and incorporation of microsensors
**Challenges**	Lack of reproducibility with natural ECM; synthetic ECM requires upregulating reagents	Generates large & heterogenous spheroids; imposes shear forces on cells	Media change is difficult; costly if robotics is involved; droplets <50 µL	Not adaptable to all cell types; heterogenous	Nanoparticles can be toxic and expensive	Selection of bioink with desired characteristics	Poor reproducibility if not automated, lack of patterning efficiency, requires expensive equipment	Low cell recovery can limit post-cell analysis
**Advantage**	Reproduces microenvironment; can observe cell adhesion and migration	Vessel size allows for a wide range of model sizes to be generated	Consistent; works with small cell population; no need for ECM; array production	Does not require ECM; cost-effective	Increased growth rates; no requirement of media or ECM	Allows for complex & organized structures; the use of multiple cell types	Allows for structure control; array production	Allows for nutrient delivery; averts necrosis; constricts model size; replicates microenvironment, array production
**Complexity**	Moderate	Moderate	Simple	Simple	Simple	Moderate	Moderate	Moderate
**Throughput**	Moderate	High	High	High	High	High	High	High
**Cost/organoid batch**	$700-800^22^	$100-200^34,99^	$2^41^	$20^47^	$200^52^	$20^69^	$1.50^17^	$~1^95^
**Volume holding organoid/organoids**	~550 µL	45 mL	<50 µL	10 mL	1 mL	40 µL	100 µL	50 µL
**Cell/organ type**	Mouse and human prostate; human ovarian, human and mouse hepatocytes; lung cancer^22–24^	Kidney; cerebral; lung^33–35^	Breast Cancer^41^	3D gastrointestinal model (epithelial and stromal cells)^47^	Mesenchymal stem cells^52^	Cardiovascular organoids^62^	Hepatocytes; hPSCs; embryonic development;^16,84–86^	Cerebral; embryoid development^91,100^

## Spheroid and organoid production techniques

### Stem cell source for organoid production

Stem cells can be classified into three groups: (i) embryonic stem cells (ESCs), (ii) induced pluripotent stem cells (iPSCs), and (iii) adult stem cells. Human ESCs can be derived from spare embryos that are not used for fertility treatments. After isolation, human ESCs can be propagated virtually to unlimited numbers while maintaining the potential to generate any differentiated cell type in the adult body, a remarkable property known as pluripotency. Similar to ESCs, iPSCs are pluripotent cells that are generated by reverting differentiated somatic cells to embryonic pluripotency through cellular reprogramming. When given the right signaling cues, both ESCs and iPSCs can be instructed to form 3D organoids from a variety of tissues such as the optic cup, liver, and brain^17–19^. In addition to their unrestricted developmental potential, iPSCs allow for cellular reprogramming of somatic cells from specific individuals to generate their genetically matched personalized organoid models. This approach holds great promise for precision medicine.

Unlike ESCs and iPSCs, adult stem cells are multipotent cells that can generate a few specialized cell types in the body. Tissue-specific adult stem cells are essential for maintaining homeostasis of the adult tissues by generating specialized cell types of that tissue. This property can be used to coax adult stem cells into forming 3D organoid models that closely resemble their tissue of origin. A notable example is single intestinal stem cells that can generate organoid models with a structure strikingly similar to that of intestinal epithelium and can be expanded in vitro indefinitely^20^. Similar adult stem cell-derived organoid models have been generated from other tissues such as mammary glands, lung, and prostate^21^. The choice of stem cells for organoid production, to a large extent, depends on the downstream applications, tissue accessibility, and expertise of the researchers. In the following sections, we will focus on current approaches for organoid production, their shortcomings and the application of microtechnology-based methods to improve organoid production (Table 1).

### ECM scaffold method

**Fig. 2:**
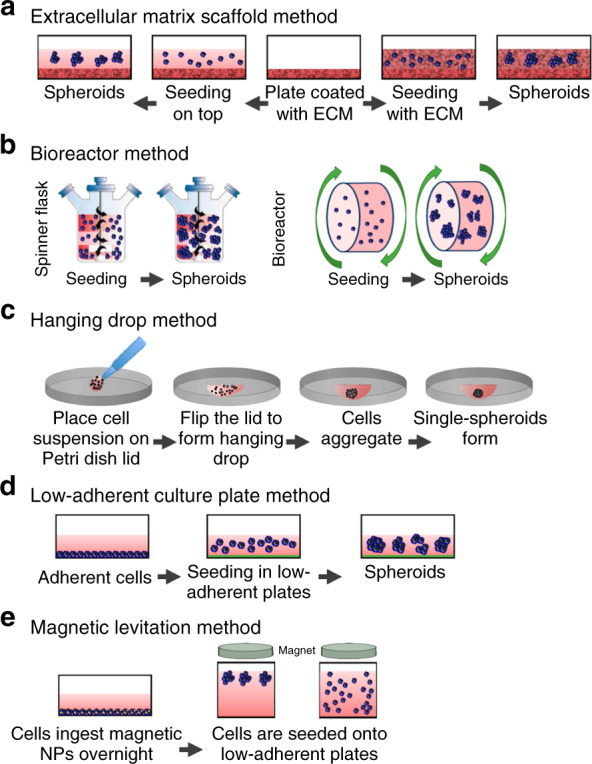
Summary of conventional organoid/spheroid production methods. A comprehensive schematic on the methods used for the generation of organoids or spheroids including (**a**) extracellular matrix scaffold, (**b**) spinning bioreactor, (**c**) hanging drop, (**d**) low-adherent cell culture plates, and (**e**) magnetic levitation method. Altered and reproduced with permission of MDPI^32^

**Fig. 3:**
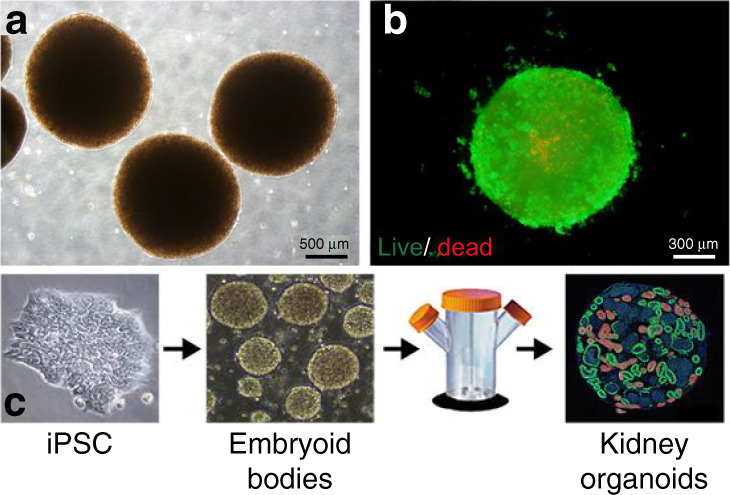
An example of liver organoids generated by the extracellular matrix scaffold method. These liver organoids were achieved by seeding hepatocarcinoma, human mesenchymal, and endothelial cells in a liver-derived 3D ECM hydrogel termed LEMgel. **a** Phase contrast and (**b**) fluorescence images show live (green) and dead (red) cells within liver organoids. Reproduced with the permission of Wiley^28^. **c** Schematic of the generation of kidney organoids using the spinning flask method. This particular example showed how embryoid bodies were formed from pluripotent stem cells and placed into the spinning flask to produce the kidney organoids. Reproduced with the permission of MDPI^34^

One of the most commonly used techniques in organoid production was developed by Hans Clever’s team. This method has been utilized to generate mouse and human prostate organoids, human ovarian tissues, and human and mouse hepatocyte organoids^22–24^. In this method, extracted adult stem cells are plated on Matrigel, a commonly used ECM protein mix, and maintained under culture conditions. When a specific cell type (basal or luminal) is desired, cells are stained with antibody, sorted with a fluorescence-activated cell sorting (FACS) system, and subsequently plated on separate Matrigel dishes. This method generates genetically and phenotypically similar organoids^22^. In these models, the fact that cell–ECM interactions drive cell organization is exploited. The ECM is usually replicated with different natural or artificial hydrogels, which include Matrigel, alginate, collagen, laminin, fibrin, and polyethylene glycol (PEG)^2,25^. In this technique, ECM agents can be plated, crosslinked, or mixed with the cell suspension. This method provides the ability to monitor cell biological processes such as cell adhesion, migration, and chemotaxis in a tissue-like setting^26^. A drawback of this method is the reproducible generation of a scaffold that represents the composition of the ECM that is naturally present in the tissue^25^. The composition of the natural hydrogel is closer to that of the in vivo ECM; however, the production of natural hydrogels is not highly reproducible. As a result, each batch of the hydrogel can have different mechanical properties, which in turn can affect and alter formation of the organoids^2^. The production of purified ECM components such as collagen and laminin is reasonably reproducible, but they do not represent the complexity of the ECM in the tissue. Synthetic hydrogels allow for more defined mechanical and biochemical properties. However, they require the addition of agents that upregulate cellular processes such as adhesion and growth^27^. Figure 2a shows a schematic of the steps involved in this ECM scaffold method. As an example (Fig. 3a, b), a mixture of hepatocarcinoma, human mesenchymal, and endothelial cells was shown to form liver organoids in a 3D liver-derived ECM hydrogel (LEMgel)^28^. To mimic the physiological ECM of the liver, LEMgel was produced by decellularization of sliced sheep’s liver. Despite the large size of the liver organoids produced in LEMgel (more than 1 mm in diameter), there is minimal cell death in this model as measured by live cell staining. In addition, organoid LEMgel promotes the expression of mature hepatocyte markers to a level closer to that of human liver when measured by quantitative RT-PCR. In another example, primary lung cancer cells were grown on agarose via the liquid overlay method^29^. In this study, cells were embedded in collagen before the formation of a 3D model of lung cancer. This approach resulted in the formation of organoids with a diameter between 50 and 200 µm. These models can be potentially useful in testing the efficacy of anticancer drugs.

### Spinning bioreactor method

Suspension cultures are an alternative three-dimensional (3D) construct method. Suspension cultures make use of incorporating agents that increase the suspension viscosity or use agitation systems. For example, the addition of carboxymethyl cellulose increases the viscosity of suspension cultures^30,31^. Spinner flasks or bioreactors are used for suspension cultures that make use of agitation to avoid cell attachment to petri dish surfaces^32^. For spinner flasks, cells are placed in a container that is constantly stirred usually by a stirring bar. Though this method allows for simple media exchange, the spheroids produced are usually large and heterogeneous in size (ranging between 65 and 300 μm)^10–13^. Bioreactors consist of rotating cell culture containers instead of stirring bars^33^. The shear force that cells experience in the bioreactor method can potentially affect cellular physiology^31^. Because bioreactors come in different sizes, spheroids of different sizes are possible. However, there is a drawback that they are heterogeneous in shape. While both spinner flasks and bioreactors induce shear forces on cells, the shear force is not as significant as in the spinner flasks^32^. Figure 2b depicts the procedure for developing spheroids using the spinning bioreactor method. As an example, Fig. 3c shows how kidney organoids are generated in bulk using spinner flasks. These kidney organoids were derived from IPSCs grown in low-attachment plates to form embryonic bodies^34^. Chemical induction of the Wnt signaling pathway in embryonic bodies resulted in the formation of kidney organoid models with varying sizes, ranging from 200 to 700 μm. Organoid models that were larger than 700 μm showed increased cellular apoptosis, suggesting that controlling organoid size is essential for promoting cellular viability in 3D culture. The simplicity of this method allows for scalable production of kidney organoids that mimic gene expression and cell biological features of the kidney in vivo. Brain tissues, referred to as cerebral organoids, were also formed using a spinning bioreactor and embedded neuroectodermal tissues in Matrigel droplets. In this method, embryonic bodies were formed from human pluripotent stem cells grown in low FGF signaling media. Embryonic bodies were induced to form 3D neuroepithelial tissues with striking similarity to the in vivo cortex, expressing markers of different cortical layers with the same spatial pattern of brain development. Neurons in the cerebral organoids showed neuronal activity, as measured by calcium imaging. This approach allowed microcephaly to be modeled by preparing cerebral organoids from iPSCs of a patient with microcephaly caused by a genetic mutation^19^. Additionally, using a rotating wall vessel, spheroids of transformed lung cells (bZR-T33) were formed over a period of several weeks, exhibiting immunostaining profiles that are similar to those of human lung tissues^35^. Spinning bioreactors allow for batch production of spheroids with a large size range.

### Hanging drop method

**Fig. 4:**
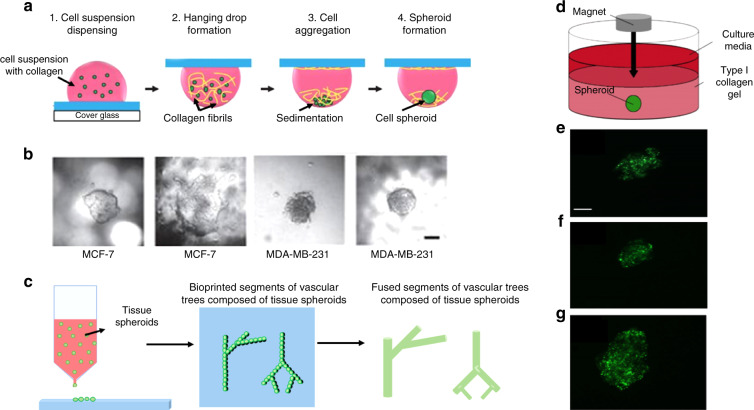
Schematic of an example of the hanging drop method. In this version of the hanging drop method, 3D spheroid culture is achieved using a PDMS-based hanging drop array (PDMS-HDA). **a** Hanging drop steps of 3D cell culture using the PDMS-HDA device. **b** Breast cancer spheroid models of MCF7 and MDA-MB-231 cells in 500 μg/ml (left) and 1 mg/ml (right) collagen-containing medium drops, respectively, were formed. Scale bar: 100 μm. Reproduced with the permission of Scientific Reports^41^. **c** General schematic of the 3D bioprinting method. Tissue spheroids are dispensed by a bioprinter in vascular tree segments and are allowed to morphologically evolve into vascular tree geometries during tissue fusion^61^. **d** Demonstration of magnetic levitation. Implementing magnetic levitation, mesenchymal stem cell (MSC) spheroids derived from cells seeded at different concentrations: (**e**) 6×10^3^, (**f**) 1×10^4^, and (**g**) 2×10^4^ cells/mL. Images show that there is a direct relationship between seeded cell concentration and spheroid size. Scale bar: 10 μm. Reproduced with permission from MDPI^52^

The hanging drop method is an air–liquid interface technique (Fig. 2c) that relies on the accumulation of cells at the liquid-air interface to form spheroids. These cells are initially suspended in a droplet of medium and placed on the back surface of a petri dish lid. Droplets are held there as a result of surface tension forces and gravity^36,37^. The suspended cells and lids are then placed on the petri dish, which contains phosphate buffered saline (PBS) to avoid the evaporation of droplets^38^. The emergence of hanging drop plates (HDPs), which create an array of spheroids in a dish, has streamlined the production of spheroids with this method^38,39^. This platform has also been combined with liquid-handling robotics, enabling the simultaneous manufacturing of a large number of 3D constructs^32^. For this method, there are several advantages including simplicity, consistency, lack of requirement of matrices, ability to upscale for high-throughput production, and ability to produce spheroids from a small population of cells. Some disadvantages of the hanging drop method involve the high costs of robotics, inability to use large liquid droplets (>50 µL), and inability to change cell culture medium without adversely affecting the spheroid^40^. An example of the hanging drop method is shown in Fig. 4a, b, where breast cancer spheroids were generated using breast cancer cell lines such as MCF7 and MDA-MB-231^41^. Two different collagen concentrations of 500 and 1000 µg/mL were used to produce the spheroids. This method can be used to compare the invasiveness of spheroids, their response to anticancer drugs and coculture effects at a large scale, making it a viable option for the generation of personalized cancer spheroid models. By comparing the effect of anticancer drugs between 2D monolayers and 3D spheroids, the authors showed that 3D spheroids are more resistant to drug treatment.

### Low-adherent cell culture plate method

The use of low-adherent or hydrophilic treated cell culture plates (Fig. 2d) has also been implemented^42,43^. In this method, plates are treated further with agents such as covalently bound neutral hydrophilic hydrogels that inhibit cell attachment, protein absorption, and enzyme activation^44,45^. This treatment causes plated cells to clump and form spheroids. This method, however, does not always form spheroids for some specific cell types, which results in additional steps to form desired cell aggregations. Similar to the hanging drop method, this method is simple to execute, allows for high throughput, and is relatively cost-effective compared to other methods^32^. This method can routinely obtain spheroids with a diameter of 370-400 µm^46^. Microscopy analysis of these spheroids can speed up anticancer drug screening by measuring the growth rate of induvial spheroids over time using phase-contrast imaging. As an example, a 3D gastrointestinal culture was derived from a mixture of epithelial and mesenchymal stromal cells in a collagen air–liquid interface system. This model was shown to proliferate and differentiate for over 60 days, which enabled replication of the microenvironment of an intestinal stem cell niche^47^.

### Magnetic levitation method

Magnetic levitation (ML, Fig. 2e) is another method for organoid production^48,49^. In this method, cells are cultured and incubated with bacteriophages, polydisperse gold nanoparticles, and magnetic iron oxide nanoparticles (<50 nm) coated with hydrogels overnight^50^. The nanoparticles are ingested by the cells, and the cells are trypsinized. Once placed in a low attachment plate, a magnetic lid is placed on top. This magnet, in turn, attracts the nanoparticles and creates a liquid-air cell suspension. Cells merge together and begin to generate ECM proteins^14,51^. This method enables faster spheroid growth rates and the replication of necrotic and hypoxic regions, and it does not require a specific medium or scaffold. However, nanoparticles can be costly and may induce toxic effects on cells if used in large quantities^32^. Regardless, this technique provides better size control of the construct. As an example (Fig. 4d–g), incorporation of magnetic nanoparticles in mesenchymal stem cells (MSCs) allows for the production of spheroid models using ML^52^. This model was used to track the migration of individual MSCs in the spheroids in response to interleukin 6, revealing mechanistic differences between 2D migration in monolayer culture and 3D migration in spheroid models. Incorporation of fluorescently labeled nanoparticles allows the migration of MSCs to be monitored using fluorescence microscopy. These nanoparticles are primarily cytoplasmic, with no noticeable cellular toxicity. However, it is unclear whether nanoparticles can cause more subtle changes in cellular functions.

### Bioprinted method

Bioprinters have recently gained much traction in the fabrication of 3D constructs. Though not low cost, this technology allows for the precise and versatile printing of multiple components, including ECM, cytokines, and cells, which enable improved reproduction of the microenvironment^46^. Bioprinters function based on additive manufacturing, which deposits the desired material layer by layer until reaching the final desired complexity in the structure. As a result, bioprinters allow the replication of complex tissues or deposition of primary cells that can then undergo histogenesis to form organized biological structures^53,54^. Using bioprinters, vascular grafts, skin, bone, and heart tissue, and cell scaffolds have all been generated^55–59^. This technology renders custom organized structures with multiple cell types and mass production capabilities^54^. Perhaps the main limitation in bioprinting is the availability of bioinks with desired characteristics and viscosity, in addition to controlling tissue development and function^53,60^. As an example (Fig. 4c), 3D bioprinting was utilized to generate vascular tree segments of tissue spheroids that, after undergoing tissue fusion, can enable organ constructs^61^. In addition, organoids were constructed by 3D printing a composite bioink with endothelial cells, followed by the addition of cardiomyocytes^62^. As a result, using this 3D bioprinting method, it was possible to generate endothelialized human myocardium from pluripotent stem cells. In this study, cardiomyocytes that are grown on bioprinted scaffolds show typical features of mature cardiomyocytes such as the expression of markers associated with contractility as well as organized sarcomeres. Cardiac tissues generated by this method start spontaneous beating after 48 h. The frequency of beating can be modulated by precisely controlling the physical properties of bioprinted scaffolds. The fact that these organoid models can be generated by using human IPSCs could pave the way for the development of personalized drug screening approaches.

## Challenges in spheroid and organoid production

### Simplicity of models

One of the challenges in organoid and spheroid production is the difficulty in reconstructing the complex in vivo cellular diversity, ECM, and signaling of organs or tumors^63^. Spheroids, for example, can establish microenvironments that allow them to generate phenotypes that exist in tissues^64^. However, spheroids are usually composed of one single cell type and are unable to completely replicate the intricate contacts with other cell types^65^. In addition, spheroids may not fully reproduce the tumor genetic heterogeneity observed in vivo^66^. Organoids, on the other hand, do self-assemble into complicated in vivo structures, but they usually lack vascularization or the mechanical stimulus of blood flow, immune cells, or stroma^67^. To improve 3D cell models, it is essential to attain the cellular diversity that exists in vivo. For example, microglia are resident innate immune cells found in the brain. However, it remains unclear to what extent the current brain organoids incorporate microglia at the level that is observed in the brain^68^. However, microglia play an extremely important role in neurological diseases such as Alzheimer’s, where they are involved in generating a neuroinflammatory agent that is critical in the pathological process of the disease^69,70^.

### Nutrient and gas delivery of models

**Fig. 5:**
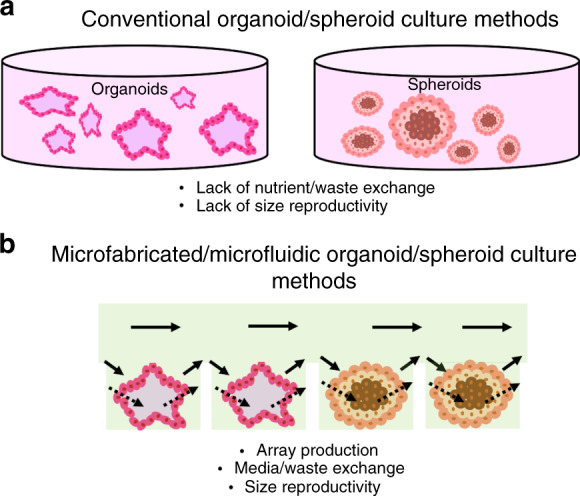
Comparison between conventional and microfluidic organoid/spheroid production methods. Diagram demonstrating the current challenges in (**a**) conventional organoid and spheroid production techniques including lack of proper nutrient delivery and exchange, as well as lack of size reproducibility. **b** Microfabricated and microfluidic-based approaches allow for array production with improved media exchange, as well as improved size control due to a defined culture area

Organoid and spheroid production requires nutrients and oxygenation for appropriate development (Fig. 5a)^71^. Proper vascularization and angiogenesis are necessary for nutrient and oxygen delivery in tumors but are difficult to reproduce in vitro in both spheroids and organoids. For example, brain organoid models can take several weeks before forming fully mature and functional neurons. The long maturation and growth of these 3D brain models hinder efficient nutrient, gas, and waste exchange. Current models are composed of embryoid bodies (EBs) encased within Matrigel and placed in petri dishes in spinning bioreactors to form organoids^72^. However, the lack of vascularization limits the development of organoids into more mature stages. This limitation is a major disadvantage, especially for organoid models of adult brain diseases or studies involving human brain aging in vitro. Introducing endothelial-lined vessels and nutrient flow would aid the elimination of necrosis that is observed in these models and allow the model to mature to larger and more complex adult-like brains^73^.

## Reproducibility of models

The most significant challenge facing organoid and spheroid production is the inability to control the size to improve the reproducibility of models (Fig. 5a)^19,74^. The lack of reproducible and standardized models is the consequence of the modest engineered cell microenvironment and ECM. It is challenging to control the size and cell numbers in these models without the incorporation of physical and scaffold geometric constraints. Differences between models inhibit mass production of the cell structures, which is critical for many applications such as accurate drug screening investigations^19^.

## Microfabrication-based solutions in spheroid and organoid production

Microfabricated organoids are those that are generated through the use of techniques and methods implemented in the development of microelectromechanical systems (MEMS). These techniques offer the benefit of high throughput and low cost in mass production. Two areas with the potential to transform organoid production are micropatterning and microfluidics.

### Micropatterning

**Fig. 6:**
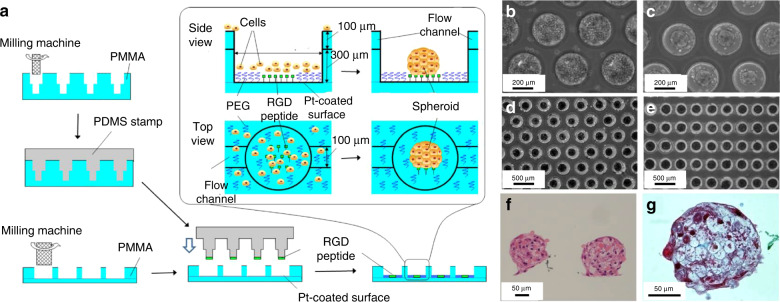
Schematic of an example of a micropatterning technique to generate arrays of spheroids. **a** A microcontact stamp was fabricated by milling a PMMA plate to form wells with 100 μm head diameter. This milled plate was ultimately used as a stamp mold onto which PDMS was casted to produce the microcontact PDMS stamp. A second PMMA plate was also milled to form wells (300 μm in diameter and 400 μm in height) that connect flow channels (100 μm wide and deep). The entire assembly (also termed the spheroid microarray chip) was then coated with a thin film of platinum, and the PDMS stamp was used to create cell attachment areas by printing 1 mM RGD peptide onto the bottom of the wells. The device was then dipped in 5 mM PEG-SH in ethanol to eliminate nonspecific cell binding around printed RGD peptide areas, resulting in spheroid production. Images show the spheroid microarray chip with (**b**) primary hepatocytes. Within 2 days of culture, (**c**) hepatocyte spheroids are observed that exhibit uniform diameter. **d** An image of the array hepatocyte spheroids within wells and those within (**e**) flow-type chips after 7 days of culture. Cross sections of hepatocyte spheroids generated with the spheroid microarray chip stained with (**f**) hematoxylin and eosin (**g**) and Masson trichrome after 3 days of culture. Reproduced with the permission of AIP publishing^16^

Typical micropatterning methods include techniques such as microcontact printing and soft-lithography methodologies^75–81^. Microcontact printing often involves the stamping of ECM proteins such as fibronectin and gelatin onto culture substrates such as glass and polystyrene. These extracellular matrices essentially become small cell adhesion sites. For example, Fig. 6 shows how a PMMA plate was micromilled to form the mold for a PDMS stamp^17^. This PDMS stamp was then used to create an array of RGD-peptide binding sites at the center of micromilled PMMA wells (Fig. 6a). After primary hepatocytes were seeded into the wells (Fig. 6a), hepatocyte spheroids formed within 2 days of culture (Fig. 6b). Figure 6c, d shows part of the array of 1500 formed spheroids within wells and wells with flow after 7 days of culture. Spheroid cell analysis with hematoxylin and eosin staining (Fig. 6e) showed viability even at the core of the ~150 µm diameter spheroids, while Masson trichrome (Fig. 6f) staining identified the collagen fibrils within the round spheroid. Micropatterned structures and collagen gels have also been used to drive intestinal epithelial cells to self-renew into human small intestinal organoids^82^. Using PDMS stamps fabricated from a standard lithography process, the architecture of rounded pillars and adjacent microwells replicated villi and crypt structures, respectively, usually observed in the native intestine. Collagen hydrogels were micromolded into tall micropillar structures, and human small intestinal cells were seeded, proliferated, and induced with a chemical gradient of growth factors (Wnt-3A, R-spondin 3 and noggin) to promote proper differentiation into human small intestinal organoids. In another study, human ESCs were micropatterned into micron-sized islands of human laminin-521 (LN-521), and treatment with BMP4 enabled the self-organizational patterns and expression markers observed in gastrulating embryos^83^. Unfortunately, if stamping is a manual process, it often results in poor reproducibility and lack of patterning efficiency. However, the clear advantage of microcontact printing is that it allows for high-throughput platforms. This method has made it possible to generate microcontact printed islands with 96-well microtiter plates^84^.

Soft lithography is another micropatterning method that allows for mass production and is more accurate and reproducible than manual microcontact printing. However, soft lithography requires the use of sophisticated and expensive equipment that has to be maintained in controlled environments such as cleanroom facilities. Photo-oxidizing PEG with deep UV light (<200 nm) has become an attractive alternative for high-throughput patterning and has been applied to the high-content screening of hPSC lines^84,85^. In addition, the application of micropatterning chips with defined sizes has facilitated the generation of 2D models of early embryonic development with reproducible sizes and shapes^86^. In this method, embryo-like structures were generated by geometrically confining pluripotent stem cells in disk-shaped laminin-coated chips and were induced to generate three distinct regions corresponding to embryonic germ layers by activation of BMP signaling. Geometrical confinement of these 2D embryo-like structures by micropatterning significantly enhances the reproducibility of the production method.

### Microfluidics

**Fig. 7:**
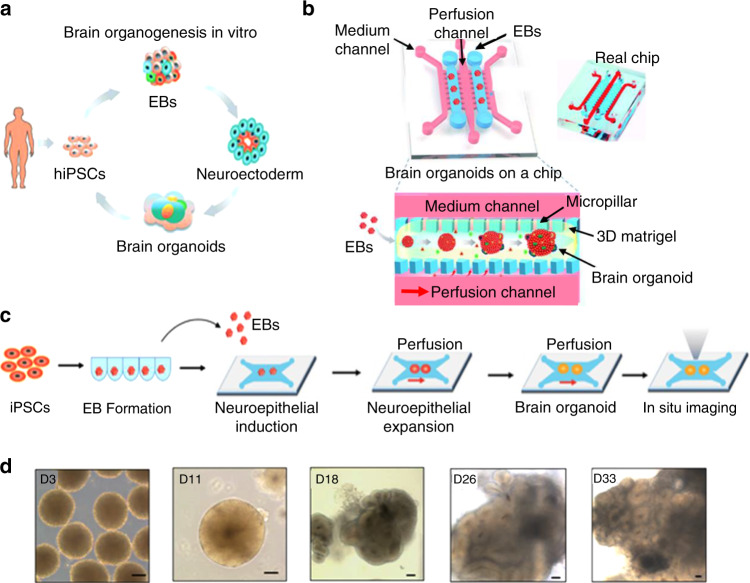
Example of a microfluidic device for brain organoid production. **a** Diagram of the in vitro brain organoid generation process from hiPSCs. **b** Schematic of the microfluidic chip used as an organ-on-a-chip device for the derivation of brain organoids where embryoid bodies result from hiPSCs and encapsulated with Matrigel. Adjacent perfusion channels were used to infuse the mixtures; these conditions allowed for the differentiation and organization of embryoid bodies into brain organoids. **c** Depiction of the process of brain organoid culture and differentiation within the microfluidic chip. **d** Images of cell organoids acquired on days 3, 11, 18, 26, and 33 of culture. Scale bars: 100 µm. Reproduced with the permission of the Royal Chemistry Society^92^

Microfluidics has also made an impact on 3D cultures due to the microenvironment that is replicated; it allows for the continuous infusion of nutrients and growth factors. Microfluidic technology also enables precise replication of cell-cell contacts, matrix characteristics, biochemical and mechanical cues, and stimuli. A simple microfluidics-based 3D cell construct usually consists of one cell type, but more complicated constructs with multiple cell types have been reported^87–89^. These multicellular microfluidic-based devices are also known as organ-on-a-chip devices. Due to their miniaturized size and arrayed microfabrication methods, microfluidic platforms can be used for high-throughput production. However, further post-cell analysis can be difficult due to the small number of cells available. Organ-on-a-chip models have shown much promise as they induce nutrient perfusion and avert necrosis. This necrosis inhibits organoid development and promotes cell death at the center of the organoid. For instance, microfluidic-based brain organoids have been shown to circumvent staggered progression, as they can develop convolutions at a particular cell density and nuclear strain^90^. After image analysis, researchers were able to deduce that the surface wrinkling and folding are attributed to the cytoskeleton shrinking at the center and nuclear stretching at the perimeter. Microfluidic chips can replicate a microenvironment where Matrigel scaffolds are present in a confined geometric space that promotes the wrinkling structure of the brain organoid while simultaneously having access to nutrient and waste exchange. As a result, these microfluidic platforms are good candidates for the replication of heterogeneous tissues, as well as the observation and investigation of biological and biophysical mechanisms in brain development. One study showed the utilization of a microfluidic chip to generate brain organoids in vitro, where ihPSCs underwent self-renewal to form embryoid bodies, then neuroectoderm and eventually organoids^91^ (Fig. 7a–c). Within this microfluidic device, embryoid bodies were mixed with Matrigel and perfused with media through adjacent channels separated by micropillar features (Fig. 7b). Matrigel allowed proper dispersal of nutrients, gas, and soluble agents and drove stem cell differentiation, while the flow media provided the culture with the necessary nutrients. Figure 7d shows the resulting brain organoids throughout the culture period of 3–33 days. In this microfluidic platform, neural differentiation and cortical structure were achieved. These organoids produced increased levels of cortical markers, which resembles in vivo cortical development. The same group also published another study showing the effects of nicotine exposure on brain development on a fetal brain organoid using a microfluidic chip^92^. This investigation showed immature neuron differentiation and sections of atypical brain development through immunohistochemical staining within the chip.

The application of microfluidics has dramatically facilitated the generation of organoid models of early human development at a scale that is not possible with conventional cell culture methods. Using microfluidics and human ESCs, it was shown that the first few days of human embryonic development could be faithfully recapitulated in vitro in a scalable and controllable manner^93^. In another published investigation, researchers harnessed the high-throughput characteristics of droplet microfluidics to generate tumor spheroids^94^. Using a flow-focusing microfluidic device composed of four inlets and one outlet, the authors were able to encapsulate MCF-7 breast tumor cells in the core and stromal fibroblast cells in the shell of alginate core-shell particles. The core tumor structure and shell stromal fibroblast cells repopulated the tumor-stroma microenvironment and provided a high-throughput drug screening method. Another organ-on-a chip device was used to produce 3D human small intestinal organoids^95^. In this device, the researchers use primary epithelial cells extracted from intestinal biopsies to derive 3D intestinal villi-like structures in situ. Within the microfluidic chip, there are two stacked chambers used as epithelial and vascular channels separated by ECM-coated membranes. Organoid fragments from external cultures are seeded within the epithelial channel. The sidewalls of the channels were fabricated and designed to mimic cyclical contraction and expansion, which replicated the peristaltic nature of the human small intestine. Cell analysis showed that epithelial cells presented barrier function and multilineage differentiation. In addition, transcriptome analysis showed that the intestine structure cultured within the chip was more similar to the human duodenum in vivo than the originally seeded organoids.

### Incorporation of microsensors

Due to the long culture periods required in organoid development, monitoring the environment and growth progression is crucial. Efforts have been made by introducing in situ biochemical, optical, and physical sensors that enable the tracking of organoid maturation. As an example, researchers developed a platform that included micro bioreactors, pneumatic valves, reservoirs, bubble traps, electrochemical and physical sensors^96^. The platform was miniaturized to fit into a benchtop incubator and utilized to monitor liver and heart organoids. The electrochemical sensor was designed to measure soluble markers produced by the organoid, and function was based on electron transfer upon a redox reaction that occurs during antibody-antigen binding. Different antibodies were functionalized on the electrode to recognize and quantify different soluble markers that were secreted. At the same time, physical sensors were implemented to measure environmental parameters such as pH, temperature, and oxygenation levels. Within the microbioreactor, cells were encased in gelatin methacryloyl (GelMA), where micropatterns were used to drive spheroid production. This model represents a good example of how microfluidics and microsensors enable the automation of spheroid and organoid generation and the possibility for applications in drug screening and toxicity.

### Cost-effectiveness

Organoid production can be expensive due to low-throughput technology and costly reagents. In doing a cost analysis, it was briefly found that organoid fabrication can cost up to $150 per organoid through traditional methods. For example, the use of a microfluidic chip to produce 24 organoids in one single chip requires 10 times less reagent volume and would decrease the cost to approximately a dollar per organoid batch (Table 1)^97^.

## Conclusions and future directions

Spheroids and organoids hold great promise in improving the replication of physiologically relevant cell and tumor models that have shed light on biological mechanisms, pathogenesis and disease treatment. These models are not only closer representations of in vivo tissues than 2D cell cultures but can also easily recapitulate human-specific biology in a dish. While several spheroid and organoid generation techniques have emerged, the current technologies for spheroid and organoid production are limited by the inability to replicate the complex cell–cell interactions, cellular diversity, and microenvironment cues of tissues in vivo, deficient nutrient and gas delivery, and lack of reproducibility. Micro-based technologies offer promising solutions to address several of the issues currently facing spheroid and organoid generation. With techniques such as micropatterning and microfluidic platforms, cell structure size and shape can be controlled. Microtechnology solutions can improve the reproducibility of spheroids and organoids, and they can be used to deliver and exchange nutrients, induce mechanical cues such as shear stress, and allow for the real-time monitoring of growth and environmental parameters through the use of sensors. In addition, microtechnology techniques lend themselves to mass production, which is required for pharmaceutical testing and commercial applications. Though microtechnology solutions have allowed for spheroid and organoid improvement, their full potential has yet to be met. The future of microtechnology-assisted spheroids and organoids will depend on how well multiple organoids can be incorporated into a single platform to reproduce the complex cell-cell microenvironment observed in vivo. Such efforts will require careful design and optimization of chambers and media. As microtechnology methods become more precise, so will 3D cell culture models. Another exciting area for future investigation is the assembly of organoids from different tissues to form functional units of the human body, also known as assembloids, which will allow us to study the interconnection of human tissues in vitro^98^. With proper sophisticated design and technological advancement, it is feasible that spheroid and organoid platforms will replace animal model studies as well as current in vitro models for applications in pathogenesis, biological mechanisms, and drug screening.

## Supplementary information

Supplementary information
