# Supplementary material for: Microtechnology-based methods for organoid models
Source: Microsyst Nanoeng. 2020 Oct 5;6:76. doi: 10.1038/s41378-020-00185-3 (PMC8433138; doi:10.1038/s41378-020-00185-3)
Supplement: Supplementary file 1 — Supplementary information [file 41378_2020_185_MOESM1_ESM.doc]

# ***Microsystems & Nanoengineering***

Organ-on-a-Chip: Micromanufacturing offers a boost to three-dimensional cell cultures

Microtechnology-based approaches could overcome the limitations of current three-dimensional cell and tissue culture processes. Complex 3D cultures provide deeper insights into human biology and pathology than standard 2D cultures, but their high complexity brings issues like low reproducibility and low throughput. In this paper, Rahim Esfandyarpour, PhD, Assistant Professor of Electrical Engineering, & Biomedical Engineering, and his team from the University of California, Irvine, Santa Cruz, and Stanford University introduce the benefits of micro-scale technologies. The team describe how micro-contact printing of cell-scaffold proteins onto culture mediums allows for higher throughput. The team also describe the construction of an “organ-on-a-chip,” where cultures are constrained between microfluidic nutrient exchange channels, driving the development of complex and accurate tissue structures. Organ-on-a-chip devices are highly economical and offer a platform that is yet to be fully exploited.

Related article manuscript number: MICRONANO-01185R

Article title: Microtechnology-based Methods for Organoid Models

Corresponding author and affiliation/s: Rahim Esfandyarpour, PhD, Assistant Professor, The University of California, Irvine, EECS, BME, Irvine, CA, United States

**About your Editorial Summary — please read**

**Before approving this Editorial Summary, please carefully check that (1) the summary text lists the correct author(s) and (2) the spelling and order of all author names and affiliations are correct.**

This **Editorial Summary** is based on your manuscript that was recently accepted for publication in *Microsystems & Nanoengineering*. It provides a non-specialist audience with a synopsis of your key research outcomes and conclusions. This value-added service provided by Springer Nature is designed to raise interest in your research across the broader community.

Springer Nature will publish the summary on the journal’s website, and it will be freely available under a under the CC BY licence (Creative Commons Attribution v4.0 International Licence) (see the journal website for details). We encourage you to re-use the summary to bring attention to your research; for example, you can host it on your own website and share it via social-networking platforms. Please attribute the summary to *Microsystems & Nanoengineering*and your article (e.g. by providing a link to your article) and do not make derivatives.

Please note that to maximise the usefulness of these summaries they must follow several stringent guidelines:
-- Spelling, punctuation and style are set according to *Nature* editorial guidelines. As this summary is aimed at non-expert readers, some concepts and technical terms will be simplified.
-- Total length must be no more than 135 words. It is likely that not all points in the paper will be covered.
-- The first sentence must be no more than 280 characters, including spaces, to allow use on microblogging sites.
-- The headline must consist of a brief generic subject identifier followed by a short description. No more than 10 words in total.

Please contact the editorial office ([mems_nano@mail.ie.ac.cn](mailto:mems_nano@mail.ie.ac.cn)) immediately with corrections should you find any factual errors in this Editorial Summary.
